# Reprogramming of Glutamine Amino Acid Transporters Expression and Prognostic Significance in Hepatocellular Carcinoma

**DOI:** 10.3390/ijms25147558

**Published:** 2024-07-10

**Authors:** Vincent Tambay, Valérie-Ann Raymond, Laure Voisin, Sylvain Meloche, Marc Bilodeau

**Affiliations:** 1 Laboratoire d’Hépatologie cellulaire, Centre de Recherche du Centre Hospitalier de l’Université de Montréal, Montréal, QC H2X 0A9, Canada; 2 Institut de Recherche en Immunologie et en Cancérologie de l’Université de Montréal, Montréal, QC H3T 1J4, Canada; 3 Département de Pharmacologie et Physiologie, Université de Montréal, Montréal, QC H3C 3J7, Canada; 4 Département de Médecine, Université de Montréal, Montréal, QC H3T 1J4, Canada

**Keywords:** liver, hepatocellular carcinoma, metabolic reprogramming, glutamine, transporters

## Abstract

Hepatocellular carcinoma (HCC) is the most prevalent primary liver malignancy and is a major cause of cancer-related mortality in the world. This study aimed to characterize glutamine amino acid transporter expression profiles in HCC compared to those of normal liver cells. In vitro and in vivo models of HCC were studied using qPCR, whereas the prognostic significance of glutamine transporter expression levels within patient tumors was analyzed through RNAseq. Solute carrier (SLC) 1A5 and SLC38A2 were targeted through siRNA or gamma-p-nitroanilide (GPNA). HCC cells depended on exogenous glutamine for optimal survival and growth. Murine HCC cells showed superior glutamine uptake rate than normal hepatocytes (*p* < 0.0001). HCC manifested a global reprogramming of glutamine transporters compared to normal liver: SLC38A3 levels decreased, whereas SLC38A1, SLC7A6, and SLC1A5 levels increased. Also, decreased SLC6A14 and SLC38A3 levels or increased SLC38A1, SLC7A6, and SLC1A5 levels predicted worse survival outcomes (all *p* < 0.05). Knockdown of SLC1A5 and/or SLC38A2 expression in human Huh7 and Hep3B HCC cells, as well as GPNA-mediated inhibition, significantly decreased the uptake of glutamine; combined SLC1A5 and SLC38A2 targeting had the most considerable impact (all *p* < 0.05). This study revealed glutamine transporter reprogramming as a novel hallmark of HCC and that such expression profiles are clinically significant.

## 1. Introduction

Hepatocellular carcinoma (HCC) is the most prevalent primary liver cancer and the fourth leading cause of cancer-related mortality worldwide [1]. Indeed, despite novel advances in cancer management with the arrival of innovative treatment modalities, such as immunotherapy, the 5-year overall survival of patients with HCC remains a bleak 20% as of 2021 [2]. Major effort is still required for a better understanding of hepatocarcinogenesis and HCC pathophysiology in order to decrease HCC-related morbidity and mortality. In 2011, the cancer cell phenotype was characterized through a revised set of broad hallmarks described by Hanahan and Weinberg, which encompass metabolic reprogramming and alterations in cellular energetics [3]. Indeed, cancer cells require optimized cell metabolism for survival within distinct microenvironments as well as for rapid cell division. This phenomenon occurring within cancer cells is of particular interest in HCC as it develops in the liver, a highly metabolically active organ.

The liver is key in regulating systemic metabolism, from glucose metabolism to protein synthesis and detoxication [4,5]. The liver is also highly involved in the metabolism of the neutral non-essential amino acid glutamine, which is the most abundant amino acid in blood [6,7]. Through its role as a nitrogen transporter, glutamine contributes to ammonia homeostasis by transporting waste nitrogen from the periphery toward the liver [8]. In hepatocytes, glutamine is used as a substrate for many pathways, such as gluconeogenesis in response to pancreatic glucagon release during hypoglycemia, as well as in protein synthesis [5,8,9]. Free ammonia released through glutamine breakdown is excreted as urea by kidneys after detoxication through the hepatic urea cycle [5,10,11]. Though glutamine is at the forefront of metabolic pathways in hepatocytes, malignant transformation leads to considerable alterations of metabolism and dependence on exogenous nutrients. As such, numerous studies have shown an important role for glutamine in cancer cell metabolism, which extends to the concept of glutamine addiction [12,13,14]. Glutamine addiction is the phenomenon in which cancer cells require environmental glutamine for survival and proper growth [12]. Hence, this increase in demand for glutamine uptake requires optimal transport of the amino acid from the tumor microenvironment. To do so, specific amino acid transporters are required, as glutamine is a polar amino acid and is restricted to secondary active transport. These constraints for glutamine flux are shared between functional hepatocytes and HCC cells. In normal hepatocytes, glutamine is transported across the plasma membrane differentially according to hepatic zonation. Whereas periportal hepatocytes have a net influx of glutamine for the subsequent metabolism and ammonia detoxication, perivenous hepatocytes principally release glutamine back into blood circulation [15,16]. In HCC, conversely, reprogramming of glutamine transporters is of great importance for tumorigenesis and cancer progression considering their dependence on exogenous glutamine. The mechanisms through which HCC cells feed themselves on nutrients, such as glucose and glutamine, is, therefore, of great interest. Glutamine transporters are members of the solute carrier (SLC) superfamily, a set of diverse proteins with characteristic substrate affinities, specificities, and transport mechanisms.

Among the various transporters capable of controlling cellular glutamine flux, SLC1A5 (ASCT2), is a sodium-dependent exchanger selective for glutamine, alanine, serine, cysteine, threonine, and asparagine [15,17]. SLC1A5 is a target gene of the c-Myc oncoprotein as well as the pRb/E2F tumor suppressor pathway, which has led to a deep interest in characterizing its role in tumorigenesis and cancer biology [15,18]. As such, SLC1A5 has been studied in various cancer types, its upregulation having been observed in various cancers [15,19]. SLC6A14 ensures the influx of all amino acids except glutamate and aspartate: in light of this broad selectivity of amino acids, including glutamine, SLC6A14 upregulation has been observed in several malignancies [15,20,21,22,23]. SLC7A6 is an obligatory exchanger between the intracellular cationic amino acids lysine, arginine, and histidine and exogenous neutral amino acids, expressed in many extrahepatic tissues [15,17]. SLC38A1 and SLC38A2, the sodium-coupled neutral amino acid transporters (SNAT) 1 and 2, are classified as system A transporters and support the sodium-dependent influx of glutamine, among other amino acids [15,17]. SLC38A2 contributes to periportal hepatocyte metabolism but has also been detected in malignancies of the stomach, colon, breast, endometrium, and pancreas [15,16,19,24,25,26,27,28]. SLC38A3 and SLC38A5 (SNAT3 and SNAT5) are responsible for system N amino acid transport, selective for the influx or efflux of glutamine, asparagine, and histidine [15,17]. In the liver, SLC38A3 contributes to glutamine flux through hepatocytes [15].

This study aimed to characterize glutamine transporter reprogramming that occurs during the malignant transformation of hepatocytes. In addition, the implication of glutamine transporter expression profiles was studied using clinical data to highlight the potential of such expression profiles as biomarkers of outcome in HCC patients. The functional implications of SLC1A5 and SLC38A2 in HCC cells were further studied by molecular and pharmacological inhibition to understand their roles in sustaining glutamine entry in HCC.

## 2. Results

### 2.1. Glutamine Is Required for Optimal Survival and Growth of Liver Cancer Cells

**Figure 1:**
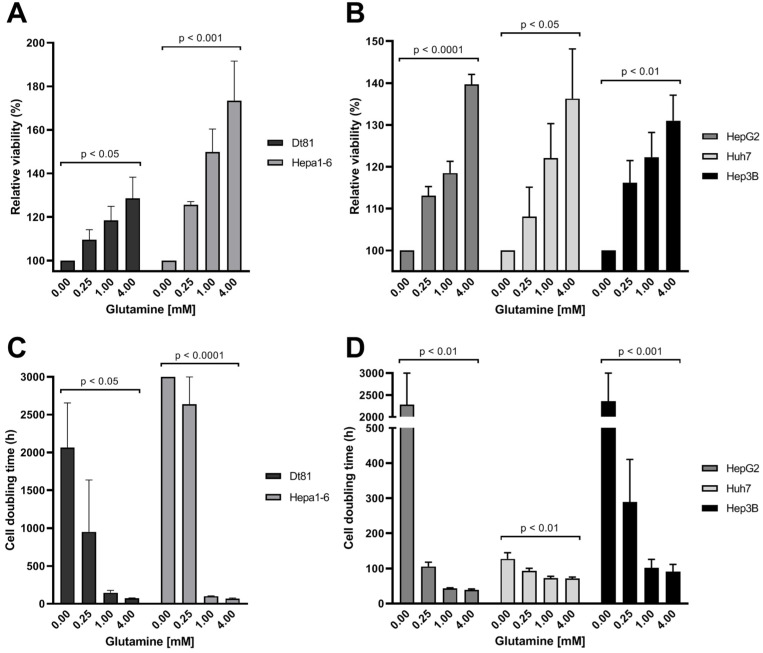
Glutamine is required for the optimal survival and growth of liver cancer cells. Viability relative to 0 mM glutamine of murine (**A**) and human (**B**) liver cancer cells after 24 h of culture with increasing exogenous glutamine up to 4 mM. Cell doubling time of murine (**C**) and human (**D**) liver cancer cells cultured with increasing exogenous glutamine up to 4 mM. All experiments were performed a minimum of *n* = 3 times, each in quadruplicate. Dt81: Dt81Hepa1-6 cells.

Cancer cells are characterized by an accelerated metabolism that responds to their state of rapid proliferation. As such, we first evaluated the influence of exogenous glutamine on cell survival and proliferation in several in vitro models of murine and human HCC. Figure 1A,B show that exogenous glutamine increased cell viability in a dose-dependent manner in all tested models. Indeed, compared to glutamine deprivation, restoration of glutamine (4 mM) alone could sustain an increase in survival by 28.6 ± 9.7% in Dt81Hepa1-6 (*p* < 0.05), 73.5 ± 18.1% in Hepa1-6 murine HCC cells (*p* < 0.001), and 39.7 ± 2.4% in HepG2 cells (*p* < 0.0001), as well as by 36.3 ± 11.9% in Huh7 (*p* < 0.05) and 31.0 ± 6.1% in Hep3B human HCC cells (*p* < 0.01). Likewise, the availability of environmental glutamine decreased the time required for cell division in all cancer cell lines (Figure 1C,D). The doubling time was decreased to below 100 h by the presence of exogenous glutamine in Dt81Hepa1-6 (*p* < 0.05), Hepa1-6 (*p* < 0.001), HepG2 (*p* < 0.01), Hep3B (*p* < 0.001), and Huh7 (*p* < 0.01) cancer cells.

### 2.2. Highly Tumorigenic Murine HCC Cells Exhibit Increased Uptake of Exogenous Glutamine

**Figure 2:**
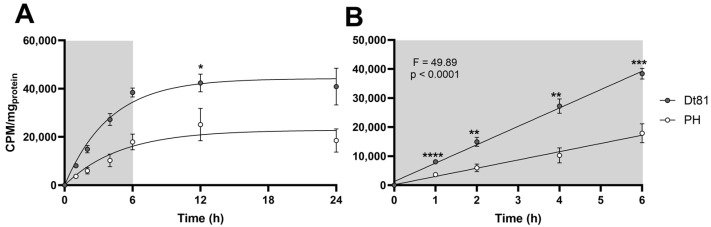
Highly tumorigenic murine HCC cells exhibit increased uptake of exogenous glutamine. (**A**) ^3^H-glutamine (20 nM) uptake kinetics over 24 h in Dt81Hepa1-6 (Dt81) HCC cells and normal primary hepatocytes (PH). (**B**) Linear phase of ^3^H-glutamine uptake in Dt81 cells and PH (0–6 h). Linear regressions of ^3^H-glutamine uptake were compared by two-tailed ANCOVA. Intracellular quantities of ^3^H-glutamine were compared between Dt81 and PH at each timepoint. Experiments were performed a minimum of *n* = 4 times, each in duplicate. *: *p* < 0.05, **: *p* < 0.01, ***: *p* < 0.001, ****: *p* < 0.0001.

Given the positive influence of exogenous glutamine on liver cancer cell survival, we hypothesized that glutamine uptake was greater in HCC cells compared to normal hepatocytes. As seen in Figure 2, glutamine uptake kinetics were significantly different between normal and neoplastic hepatocytes. Both cell types showed an initial linear phase of ^3^H-glutamine uptake from 0 through 6 h (Figure 2B), which plateaued between 6 and 24 h. The rate of glutamine uptake, however, was significantly greater in Dt81Hepa1-6 cells than in primary hepatocytes. As shown in Figure 2, after 4 h of glutamine uptake, the intracellular levels of ^3^H-glutamine in Dt81Hepa1-6 cells was 2.65-fold higher than in normal hepatocytes (*p* < 0.001). Through the linear phase, the rate of glutamine uptake was significantly greater in Dt81Hepa1-6 cells than primary hepatocytes (F = 49.89, *p* < 0.0001): Dt81Hepa1-6 cells had an entry rate of 6619.4 CPM/mg_protein_/hour in comparison to 2880.0 CPM/mg_protein_/hour in primary hepatocytes (Figure 2B). Within the plateau phase, at 24 h after onset of culture with ^3^H-glutamine, Dt81Hepa1-6 had consumed 40,858.2 ± 5371.9 CPM/mg_protein_ of ^3^H-glutamine compared to 18,501.3 ± 4162.4 CPM/mg_protein_ in primary hepatocytes.

### 2.3. Murine HCC Is Characterized by the Reprogramming of Glutamine Transporter mRNA Levels

**Figure 3:**
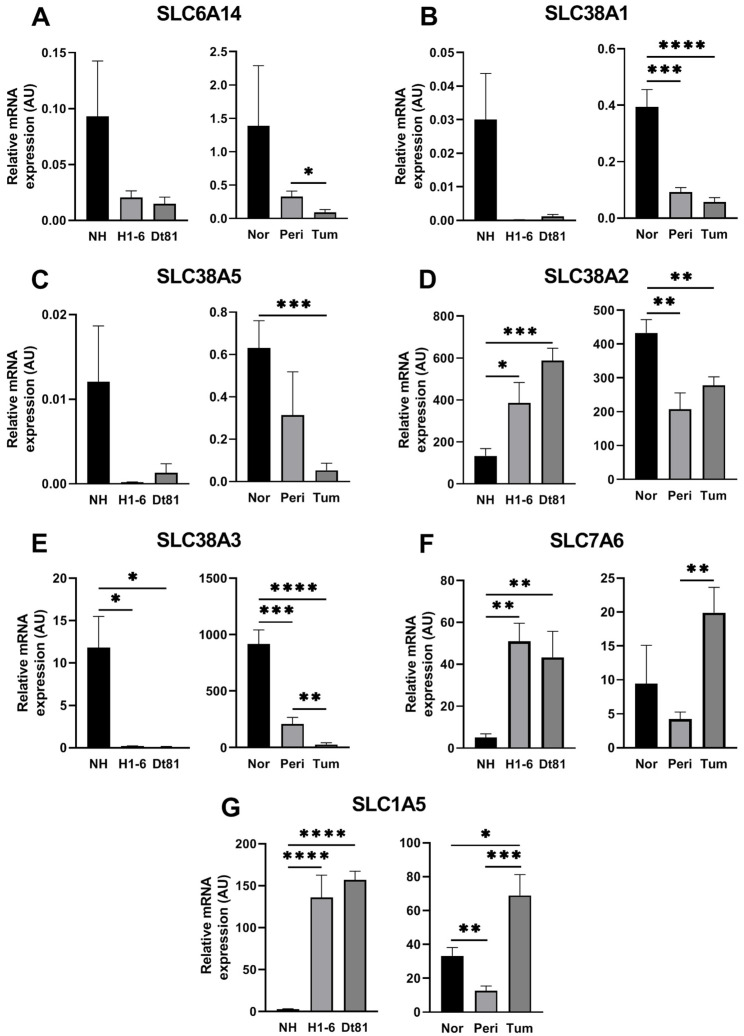
Murine HCC is characterized by the reprogramming of glutamine transporter mRNA levels. mRNA expression of glutamine transporters SLC6A14 (**A**), SLC38A1 (**B**), SLC38A5 (**C**), SLC38A2 (**D**), SLC38A3 (**E**), SLC7A6 (**F**), and SLC1A5 (**G**) relative to housekeeping genes (PPIA, HPRT1, and H2AFZ), measured using qPCR analyses. For cell cultures, mRNA was extracted after 24 h. NH: normal primary murine hepatocytes, H1-6: Hepa1-6 murine HCC cells, Dt81: Dt81Hepa1-6 murine HCC cells. Nor: normal mouse liver controls, Peri: peritumoral tissue, Tum: murine HCC tumor. AU: arbitrary units. *: *p* < 0.05, **: *p* < 0.01, ***: *p* < 0.001, ****: *p* < 0.0001.

The contrasting glutamine uptake kinetics observed between normal hepatocytes and murine HCC cells suggested that glutamine transporters could be altered in HCC. In this study, we chose Dt81Hepa1-6 cells and the parental cell line Hepa1-6 in a complementary manner, given that Dt81Hepa1-6 cells were subsequently used to study in vivo HCC in mice, the latter being much more tumorigenic than Hepa1-6 cells. As such, the mRNA expression levels of 7 glutamine uptake transporters were measured in our murine model of HCC (Figure 3). SLC6A14 and SLC38A5 were weakly expressed in all in vivo and in vitro samples, in both normal and HCC sample types (Figure 3A,C). SLC38A1 was virtually absent in all cellular samples and was, though weakly expressed in tissue samples, greater in the control liver compared to both the peritumoral liver and tumoral tissues (both *p* < 0.001, Figure 3B). Despite the SLC38A2 transporter being highly expressed in all sample types, it was particularly higher in Hepa1-6 cells compared to primary hepatocytes (*p* < 0.05), as well as in Dt81Hepa1-6 cells compared to primary hepatocytes (*p* < 0.001, Figure 3D). As seen in Figure 3D, in vivo HCC and adjacent liver were characterized by a significant decrease in SLC38A2 expression compared to the healthy liver (both *p* < 0.01). SLC38A3 mRNA was consistently downregulated in both in vitro and in vivo murine HCC models (Figure 3E). For example, SLC38A3 levels were higher in peritumoral liver than in adjacent tumors (*p* < 0.01). SLC7A6 and SLC1A5 transporters were significantly increased in HCC cells and tissue samples. Indeed, in Hepa1-6 and Dt81Hepa1-6 cell cultures, SLC7A6 levels were significantly greater than in primary hepatocytes (both *p* < 0.01, Figure 3F). Its expression within tumors was similarly increased compared to the adjacent liver (*p* < 0.01). Finally, SLC1A5 was significantly upregulated in Hepa1-6 and Dt81Hepa1-6 cells compared to hepatocytes (both *p* < 0.0001, Figure 3G). These findings correlated with what occurred in vivo, as depicted in Figure 3G, in which murine tumors had a significantly upregulated expression of 68.99 ± 12.28 arbitrary relative units (AU) compared to both peritumoral (12.70 ± 2.73 AU, *p* < 0.001) and control liver tissues (33.27 ± 4.98 AU, *p* < 0.05).

### 2.4. Glutamine Transporter Reprogramming in Human HCC Cells

**Figure 4:**
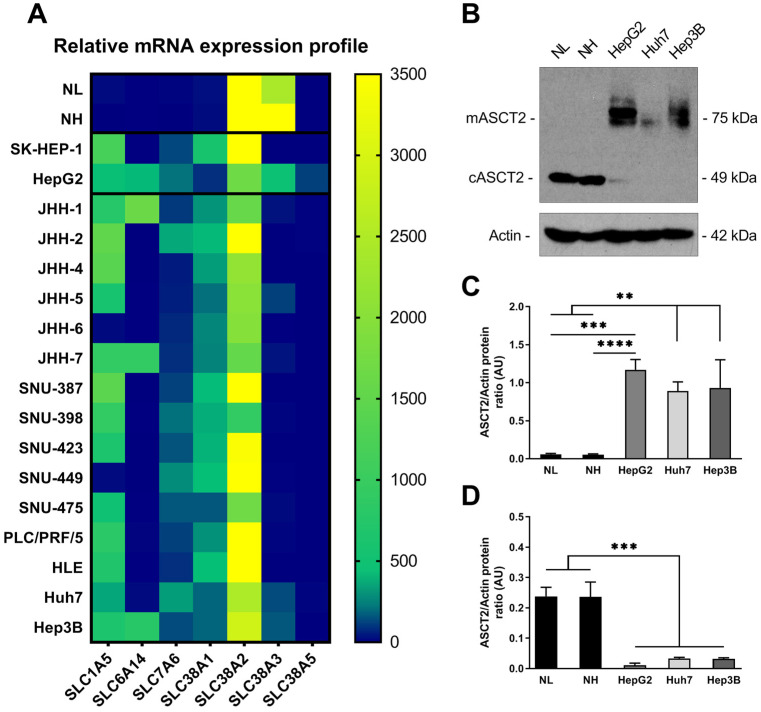
Glutamine transporter reprogramming in human HCC cells. (**A**) mRNA expression levels of the glutamine transporters SLC1A5, SLC6A14, SLC7A6, SLC38A1, SLC38A2, SLC38A3, and SLC38A5 relative to housekeeping genes (S9 and HMBS) in normal human liver tissue (NL), normal human hepatocyte isolates (NH), HepG2 and SK-HEP-1 non-HCC liver cancer cells, and a panel of 15 HCC cell lines, measured using qPCR analysis. (**B**) Representative Western blot image of ASCT2 protein expression in NL, NH, as well as HepG2, Huh7, and Hep3B cells after 24 h of cell culture. mASCT2: glycosylated surface membrane-bound ASCT2 (75 kDa), cASCT2: non-glycosylated cytosolic ASCT2 (49 kDa). (**C**) Quantification of mASCT2 (75 kDa) relative to actin. (**D**) Quantification of cASCT2 (49 kDa) relative to actin. AU: arbitrary units. **: *p* < 0.01, ***: *p* < 0.001, ****: *p* < 0.0001.

In light of the alteration in glutamine transporters that was observed in our murine HCC model, we next studied transporter expression reprogramming in human HCC. To do so, we screened a panel of 15 HCC cell lines to establish glutamine transporter transcript profiles of a diverse set of HCC cell line models as well as to identify a global trend in transporter expression in human HCC compared to normal liver and hepatocytes. Figure 4A depicts a heatmap of the mRNA expression levels of normal liver tissue, isolated hepatocyte pellets, non-HCC HepG2 (hepatoblastoma), SK-HEP-1 (endothelium-derived hepatic adenocarcinoma) cancer cell pellets, as well as HCC JHH-1, JHH-2, JHH-4, JHH-5, JHH-6, JHH-7, SNU-387, SNU-398, SNU-423, SNU-449, SNU-475, PLC/PRF/5, HLE, Huh7, and Hep3B cell pellets. In normal hepatocyte pellets, SLC38A2 and SLC38A3 were the two mainly expressed transporters, which corresponded to the results observed in whole-liver tissue extracts. SLC38A5 was nearly undetectable in all samples, with the exception of HepG2 cells. The SLC6A14 transporter, absent in controls, was expressed exclusively in HepG2, JHH-1, JHH-7, and Hep3B liver cancer cell lines. All cancer cell lines expressed high levels of SLC38A2. Interestingly, SLC38A3 expression was consistently downregulated in all cancer cells. Even in HCC cells that showed detectable levels of SLC38A3 mRNA, such as Huh7 and Hep3B, there was a minimum decrease in expression of nearly 31-fold compared to normal hepatocyte pellets. Conversely to SLC38A3, the SLC7A6 and SLC38A1 transporters were broadly expressed in all cancer cell types. SLC1A5 was the most importantly upregulated glutamine transporter in HepG2, SK-HEP-1, and in all HCC cancer cell lines with the exception of JHH-5 and SNU-449. Indeed, JHH-5 and SNU-449 only exhibited a 10- and 11-fold increase in SLC1A5 mRNA compared to control pellets, respectively, whereas upregulation of the transporter was 191-fold in Huh7 cells and up to 927-fold in JHH-2 cells. As SLC1A5 was the most considerably upregulated glutamine uptake transporter in our panel of HCC cells, we further evaluated its expression at the protein level. In Figure 4B,C, Western blot analysis revealed that glycosylated (75 kDa) ASCT2 was weakly expressed in normal liver extracts and hepatocyte isolates; on the other hand ASCT2 protein levels relative to Actin were significantly higher in liver cancer HepG2 (1.17 ± 0.13, both *p* < 0.001), Huh7 (0.89 ± 0.12, both *p* < 0.01), and Hep3B (0.93 ± 0.37, both *p* < 0.01) cells. Interestingly, normal liver and hepatocytes expressed non-glycosylated (49 kDa) SLC1A5/ASCT2 proteins at levels significantly greater than cancer cells (all *p* < 0.001, Figure 4D).

### 2.5. Glutamine Transporter Expression Profiles Are Associated with Differential Overall Survival of HCC Patients

**Figure 5:**
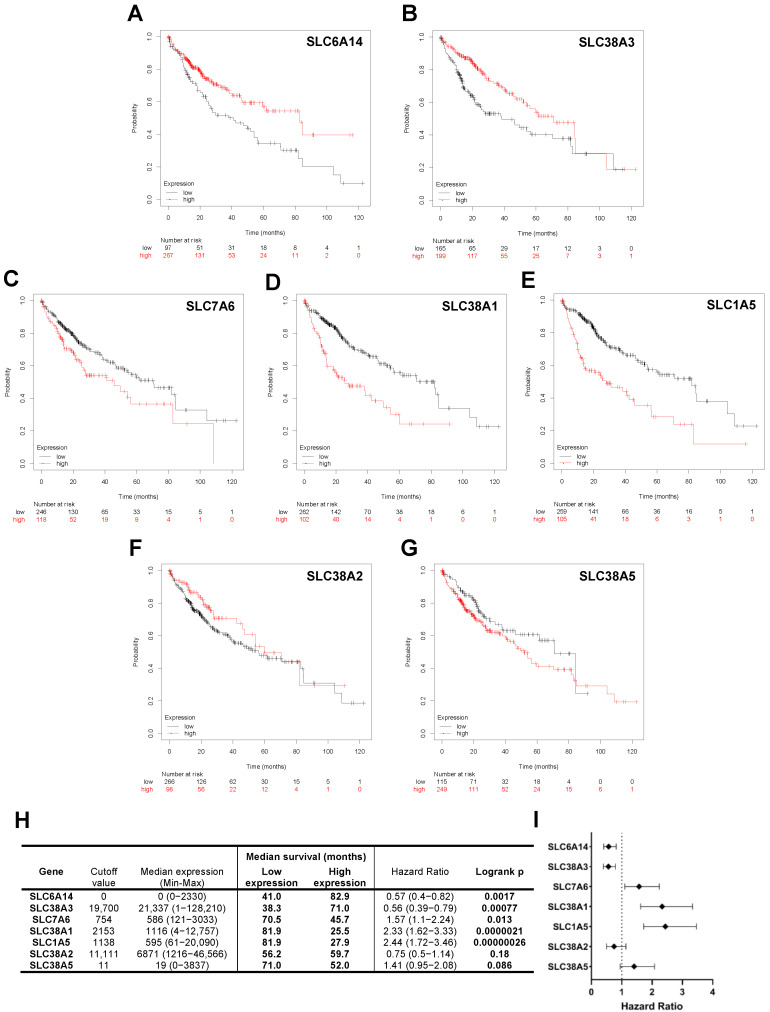
Glutamine transporter expression profiles are associated with differential overall survival of HCC patients. Analysis of overall survival within TCGA-LIHC HCC patient cohort between low and high intratumoral mRNA expression (RNAseq data) of glutamine transporters SLC6A14 (**A**), SLC38A3 (**B**), SLC7A6 (**C**), SLC38A1 (**D**), SLC1A5 (**E**), SLC38A2 (**F**), and SLC38A5 (**G**). (**H**) Summary table of cohort data, including RNAseq expression data, median survival, and log-rank analysis between low and high expression cohorts for each TCGA-LIHC gene dataset. Appropriate cutoff mRNA values were determined between lower and upper quartiles. (**I**) Forest plot depiction of log-rank hazard ratios comparing risk of overall mortality in high-expressing patients to low-expressing patients.

We further aimed to determine if the level of glutamine transporter expression had prognostic value on the overall survival of HCC patients, and as such, if specific transporters were associated with greater tumor aggressiveness and patient mortality. Complete absence of SLC6A14 mRNA, as depicted in Figure 5A, was associated with a 41.9-month decrease in the overall survival of HCC patients compared to patients having detectable tumor SLC6A14 mRNA (*p* < 0.01, HR = 0.57 (0.4–0.82)). Similarly to SLC6A14, loss of intratumoral SLC38A3 mRNA was associated with a 38.3-month median survival outcome of HCC patients, which was significantly less than those highly expressing SLC38A3 at 71.0 months (*p* < 0.001, HR = 0.56 (0.39–0.79), Figure 5B). Overexpression of SLC7A6 within HCC tumors was associated with a median 45.7-month overall survival of patients compared to 70.5 months in the low-expression cohort (*p* < 0.05, HR = 1.57 (1.1–2.24), Figure 5C). Likewise, in Figure 5D, patients having a tumor SLC38A1 expression level above the cutoff value of 2153 (median = 1116, min. = 4, max. = 12,757) had an overall survival of 25.5 months, whereas those with low expression had an overall survival of 81.9 months (*p* < 0.0001, HR = 2.33 (1.62–3.33)). Lastly, as observed in Figure 5D, patients whose intratumoral expression of SLC1A5 was considered high (cutoff = 1138 (median = 595, min. = 61, max. = 20,090)) had an overall survival of 27.9 months, which was significantly shorter than those with low intratumoral SLC1A5 with a median overall survival of 81.9 months (*p* < 0.0001, HR = 2.44 (1.72–3.46)). The tumor expression profiles of SLC38A2 and SLC38A5 were not associated with different survival outcomes within this patient cohort.

### 2.6. SLC1A5 and SLC38A2 Are Significant Contributors to Glutamine Uptake in Human HCC Cells

**Figure 6:**
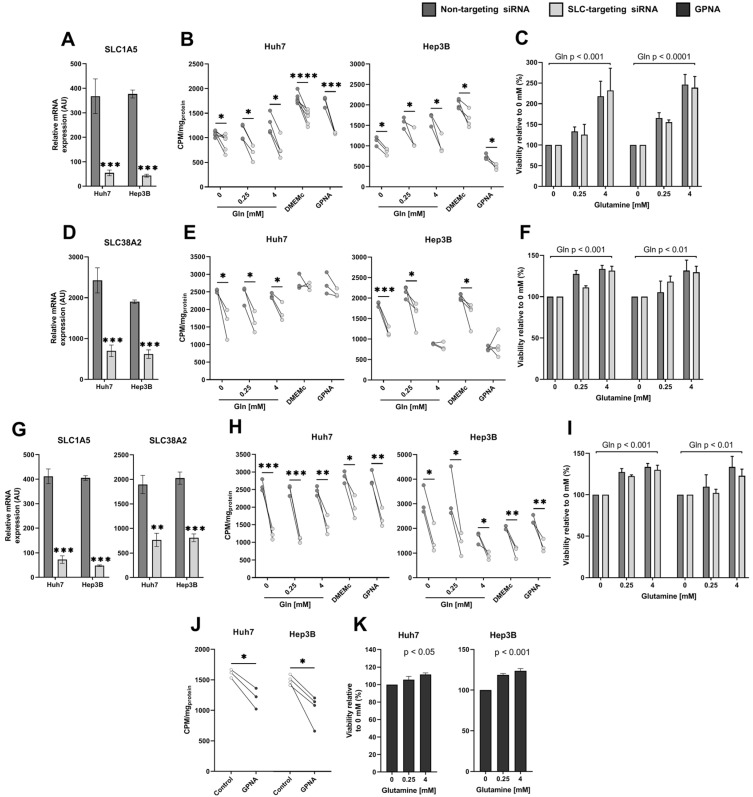
SLC1A5 and SLC38A2 are significant contributors to glutamine uptake in human HCC cells. siRNA transfection was performed to target SLC1A5 (**A**–**C**), SLC38A2 (**D**–**F**), or in combination (**G**–**I**). Validation of siRNA targeting was performed by measuring relative mRNA levels of targets SLC1A5/ASCT2 (**A**), SLC38A2/SNAT2 (**D**), or both (**G**). Intracellular uptake of radiolabeled glutamine (gln), ^3^H-glutamine (20 nM), over one hour was measured after cell conditioning with no additional gln (0 mM), low gln (0.25 mM), high gln (4 mM), DMEMc (gln-rich, glucose-rich medium), and gamma-p-nitroanilide (GPNA) treatment in DMEMc [0.5 mM], for SLC1A5 inhibition (**B**), SLC38A2 inhibition (**E**), or dual inhibition (**H**). Glutamine-dependent cell viability was compared between non-targeting siRNA control-treated Huh7 and Hep3B cells and those subject to SLC1A5 (**C**), SLC38A2 (**F**), or combined (**I**) inhibition. Intracellular uptake of radiolabeled glutamine (gln), ^3^H-glutamine (20 nM), over one hour was also measured under gamma-p-nitroanilide (GPNA) treatment [0.5 mM] compared to control (DMEMc) (**J**). Glutamine-dependent cell viability was assessed under GPNA treatment [0.5 mM] in Huh7 and Hep3B cells (**K**). AU: arbitrary units, CPM: counts per minute. All experiments were performed a minimum of *n* = 3 times, each in duplicate. *: *p* < 0.05, **: *p* < 0.01, ***: *p* < 0.001, ****: *p* < 0.0001.

The SLC1A5 transporter was significantly upregulated in several HCC cell models and associated with decreased overall survival in HCC patients. Therefore, we further aimed to elucidate the impact of its inhibition on the uptake of glutamine as well as survival and proliferation of human HCC cells. Furthermore, although SLC38A2 was identified as being significantly increased in HCC, it was the most highly expressed glutamine transporter in the studied HCC models, both murine and human. Hence, its contribution to glutamine consumption in HCC was also evaluated. Depletion of SLC1A5 or SLC38A2 expression was performed by transfection of distinct siRNA pools targeting four distinct sequences within SLC1A5 or SLC38A2 transcripts, in Huh7 and Hep3B cells, two well-characterized human HCC cell lines. Combined depletion was also performed by exposing HCC cells to both SLC1A5 and SLC38A2 siRNA pools. In addition, we used the small-molecule inhibitor GPNA to pharmacologically inhibit the activity of both transporters [29,30]. SLC1A5 expression was successfully knocked down in Huh7 and Hep3B HCC cells at the mRNA (both *p* < 0.001, Figure 6A,G) for both isolated and combined siRNA experiments. Expression of SLC38A2 mRNA was also significantly depleted in HCC cells by treatment with siRNAs alone (both *p* < 0.001, Figure 6D) and combined (both *p* < 0.01, Figure 6G).

We first measured the impact of transporter inhibition of glutamine uptake in HCC cells. As depicted in Figure 6B, SLC1A5 knockdown significantly decreased ^3^H-glutamine uptake in all studied conditions, regardless of glutamine addition, presence of glucose, or inhibition with GPNA, in both Huh7 (0, 0.25, and 4 mM gln: *p* < 0.05; DMEMc: *p* < 0.0001; GPNA: *p* < 0.001) and Hep3B (all *p* < 0.05) HCC cells. SLC38A2 knockdown also negatively impacted ^3^H-glutamine uptake in HCC cells, but most notably in conditions of low extracellular glutamine (Figure 6E). In Huh7 cells, loss of SLC38A2 decreased glutamine uptake without or with 0.25 or 4 mM glutamine supplementation (all *p* < 0.05), but not in glucose-replenished nor GPNA treatment conditions. In Hep3B cells, SLC38A2 inhibition in the absence of glutamine supplementation had the most significant effect on ^3^H-glutamine uptake decrease (*p* < 0.001), whereas cells with low supplementation of glutamine or glucose-repleted conditions also had a decreased uptake (both *p* < 0.05). As shown in Figure 6H, combined siRNA inhibition of SLC1A5 and SLC38A2 had the most considerable negative impact on ^3^H-glutamine uptake in HCC cells, decreasing uptake capacities in all conditions by nearly half in Huh7 cells (0, 0.25 mM gln: *p* < 0.001; 4 mM gln, GPNA: *p* < 0.01; DMEMc: *p* < 0.05), similarly to what was observed in Hep3B cells (0, 0.25, 4 mM gln: *p* < 0.05; DMEMc, GPNA: *p* < 0.001). In addition, GPNA treatment alone decreased glutamine uptake from 1605.0 ± 39.5 to 1201.8 ± 98.7 CPM/mg_protein_ in Huh7 cells and from 1517.6 ± 39.3 to 1038.0 ± 125.0 CPM/mg_protein_ in Hep3B cells (both *p* < 0.05). The inhibition of ^3^H-glutamine uptake by perturbing SLC1A5 and/or SLC38A2 expression genetically, or by inhibiting their activity with GPNA independently of siRNA, though significant, did reveal residual uptake activity in HCC cells (Figure 6B,E,H,J).

Given the residual uptake of glutamine under SLC1A5 and SLC38A2 inhibition, we measured the glutamine-dependent cell viability of Huh7 and Hep3B cells after RNA interference of SLC1A5 and/or SLC38A2 (Figure 6C,F,I), as well as after GPNA treatment (Figure 6K). As shown in Figure 6C,F,I, regardless of SLC1A5, SLC38A2, or their combined siRNA-mediated inhibition, Huh7 and Hep3B HCC cells maintained their capacity to utilize exogenous glutamine for the optimization of cell survival in a dose-dependent manner (Huh7: all *p* < 0.05; Hep3B: SLC1A5 siRNA: *p* < 0.001, SLC38A2 and combined siRNA: both *p* < 0.01). Furthermore, RNA interference did not negatively impact cell viability in any glutamine supplementation condition (Figure 6C,F,I), nor in DMEMc with or without combined GPNA treatment (Figure S1). Even under GPNA treatment, HCC cells were capable of using exogenous glutamine for cell survival (Huh7: *p* < 0.05; Hep3B: *p* < 0.001, Figure 6K).

The sustained ability for HCC cells to optimize survival from exogenous glutamine led us to investigate the expression of alternative glutamine transporters, which could explain this residual, significant, uptake of glutamine by HCC cells (Figure S2). The only significantly upregulated transporter in response to glutamine transporter inhibition was SLC38A5, which increased in Huh7 cells exposed to combined SLC1A5/SLC38A2 siRNA treatment (*p* < 0.05, Figure S2C). In Hep3B cells, SLC1A5 knockdown induced a decrease in SLC7A6 mRNA (*p* < 0.05, Figure S2A), whereas that of SLC38A2 led to a modest decrease in mRNA levels of SLC1A5 (*p* < 0.05) and SLC7A6 (*p* < 0.01) as well as a decrease in the glutamine-metabolizing enzymes glutamine synthetase (*p* < 0.01) and glutaminase 1 (*p* < 0.05, Figure S2B).

## 3. Discussion

Recently, metabolic reprogramming has emerged as a major phenotype of cancer cells [3,31]. Alterations in cell metabolism and energetics are acquired through diverse pathways, from the activation of oncogenic driver genes to cellular adaptations to the microenvironment [32]. These alterations are broad, as they may implicate a wide variety of metabolic pathways, for example amino acid metabolism and glycolysis [33]. Such a phenomenon is all the more pertinent in the context of HCC, as the liver is a major actor in systemic metabolism in physiology [4,5]. Given the central role of glutamine in various metabolic pathways, there is growing interest regarding its implication in cancer cell metabolism [13]. Furthermore, because glutamine is a polar, neutral amino acid, its uptake requires active transport by specific amino acid transporters. Although there is no specific transporter protein for glutamine influx or efflux through cells, select members of the solute carrier superfamily have the ability to transport glutamine into the cytoplasm from the extracellular environment [15,17]. In this study, we aimed to elucidate the expression profiles of glutamine transporters in hepatocellular carcinoma compared to those of normal liver and hepatocytes. We focused on the amino acid transporters SLC1A5, SLC6A14, SLC7A6, SLC38A1, SLC38A2, SLC38A3, and SLC38A5 by investigating their transcriptional expression profiles in HCC, their role in cellular glutamine transport function, as well as their clinical significance in HCC patients.

Thus far, cancer cell dependence on exogenous glutamine has been reported in models of various cancer types, such as glioma and lung carcinoma [34,35]. In light of these observations in other cancers, we aimed to understand if environmental glutamine was used to sustain the survival and growth of diverse HCC cell lines, of both murine and human origins. Interestingly, we consistently observed an improvement in cell viability with increasing concentrations of exogenous glutamine for all tested cell lines, including Hep3B and Huh7 human HCC cells, as well as highly tumorigenic murine Dt81Hepa1-6 cells. This dependency on environmental glutamine translated to improved proliferative ability of HCC cells in vitro. Conversely to Jin et al., who stated that Huh7 cells were glutamine-independent [36], we found that Huh7 cells utilized exogenous glutamine to optimize both survival and proliferation. Such contradictory findings may arise from varying cell culture periods: as glutamine is consumed over time by cancer cells, culture medium becomes depleted of nutrients including glutamine, in addition to increasing cell confluency limiting optimal cellular activity in vitro. Though Huh7 cell growth was the least impacted by glutamine deprivation, the rate of proliferation was still significantly increased when cells were cultured with glutamine. Together, these findings suggest that glutamine uptake from the cellular environment is relevant in HCC and that there exists a fundamental role for glutamine in the metabolic landscape of HCC.

As we observed that exogenous glutamine was used by HCC cells to optimize both cell survival and proliferation, we measured ^3^H-glutamine uptake in normal primary murine hepatocytes compared to Dt81Hepa1-6 murine HCC cells. Though normal hepatocytes did exhibit glutamine uptake in the cell culture, Dt81Hepa1-6 cells had an increased uptake of glutamine. In normal hepatocytes, namely in periportal zones of the liver acinus, glutamine influx is known to be physiologically important, as glutamine is metabolized to sustain urea cycle metabolism as well as protein synthesis and the glucose pathway [5,8,9]. Interestingly, we observed that the uptake of glutamine in HCC cells surpasses that in normal hepatocytes, which concords with its positive survival and proliferative effect on liver cancer cells. This significant increase in glutamine uptake in neoplastic hepatocytes compared to their normal counterparts prompted us to further characterize amino acid transporters that have the ability to uptake extracellular glutamine in HCC. In our murine model of hepatocarcinogenesis, SLC6A14, SLC38A1, and SLC38A5 were the most weakly expressed, suggesting that they play a minor role, if any, in glutamine flux in this model. Greater variability in these transporters was observed in our panel of human HCC cells, which better reflect HCC heterogeneity, with all cell lines having been isolated from distinct individuals’ tumors with different genetic makeups. Unlike what we observed in murine HCC, SLC38A1 was globally expressed in all human cancer cells, alongside SLC38A2 being the most highly expressed glutamine transporter in all studied HCC cells. The detection of abundant SLC38A2 in normal liver and hepatocytes also confirmed its role as a fundamental amino acid transporter for hepatic physiology [15,16]. This maintained expression of SLC38A2 together with enhanced SLC38A1 levels in HCC suggest a major role for system A transport in amino acid influx and metabolism. System A transport could be a preferred uptake mechanism for glutamine in liver cancer cells as it does not compromise the intracellular reserve of amino acids through obligatory exchange. Indeed, SLC38A1 and 2 have been described as neutral amino acid “loaders” in cancer cells, thus allowing rapid and direct influx of specific amino acids, such as glutamine [37]. Unlike SLC38A2, the loss of SLC38A3 expression did discriminate HCC from normal liver and hepatocytes. As part of system N amino acid transport, SLC38A3 is known to be expressed in normal liver [15,16]. Also, in esophageal squamous cell carcinoma, copy number alterations have been found to be the leading cause of SLC38A3 downregulation, which in turn was associated with increased epithelial-to-mesenchymal transition and tumor aggressiveness [38]. Given this possible protective role against tumorigenesis and cancer progression, loss of SLC38A3 expression might represent a pro-tumorigenic mechanism, in addition to reflecting the dedifferentiation process that inevitably occurs during HCC tumorigenesis.

Furthermore, SLC7A6 and, most importantly, SLC1A5 were the two main transporters overexpressed in all studied HCC models, both in vitro and in vivo. Within our panel of human HCC cell lines, SLC1A5 was the most highly upregulated gene in 13 of 15 cell types compared to normal liver and hepatocytes. Surface expression of ASCT2 was specific to cancer cells compared to normal liver and hepatocytes, as the 75 kDa ASCT2 protein form represents glycosylated proteins, a post-translational modification required for its trafficking to the membrane [39]. Interestingly, SLC7A6 and SLC1A5 have been shown to be targets of driver genes implicated in tumorigenesis, such as c-Myc in breast cancer, lung small cell carcinoma, colorectal carcinoma, glioblastoma, and HCC [40,41,42,43,44,45]. In TCGA methylation profiling data, *SLC1A5* promoter hypomethylation is associated with *TP53* mutation in HCC [46,47]. The pRb tumor suppressor has also been previously shown to negatively regulate SLC1A5 expression [48]. Altogether, though there exists heterogeneity between HCC cell types, a global reprogramming of glutamine transporters is synonymous with neoplastic transformation of hepatocytes. In HCC, there is a heterogeneous enhancement of several amino acid transporters, including SLC6A14, SLC38A1, and importantly SLC1A5 and SLC7A6, with a consistent decrease in the expression of SLC38A3.

To assess the clinical relevance of these observations, we performed a comparative analysis of intratumoral glutamine transporter mRNA expression levels and patient overall survival within the TCGA-LIHC cohort. As glutamine transporter expression profiles were significantly altered in murine HCC and human HCC cell lines compared to controls, we herein aimed to identify transporter genes as potential prognostic biomarkers of overall patient survival. The lack of correlation between HCC patient prognosis and intratumoral levels of SLC38A2 might be explained from its shared abundance in normal hepatocytes as well as HCC cells. The absence of SLC6A14 within patient tumors was associated with shorter patient survival rates, suggesting that SLC6A14 is expressed specifically in less aggressive HCC tumor subtypes. Similarly, loss of SLC38A3 within tumors was a characteristic of patients with bleaker prognoses. As dedifferentiation of cancer cells is globally accepted to be a hallmark of advanced malignant tumors, this supports the idea that the loss of SLC38A3 is associated with more aggressive HCC behavior. Conversely to SLC38A3, upregulation of SLC38A1, SLC7A6, and SLC1A5 transporters in HCC tumors was associated with decreased overall patient survival, with SLC1A5 being the most significantly associated with tumor aggressiveness. These select members of the SLC superfamily could be highly implicated in amino acid metabolism, and more specifically in the glutamine pathway, during HCC progression. Overall, mRNA expression profiles of specific glutamine transporters are clinically significant as they have the ability to stratify HCC patients at greater risk of mortality.

Our comprehensive study of glutamine transporters expression levels in HCC as well as their respective involvement in patient overall prognosis identified SLC1A5 as being an upregulated glutamine transporter specifically in aggressive HCC tumor subtypes. As this phenomenon had already been observed within the cancer cell metabolic phenotype, this has led to the development of glutamine transport inhibitors. Unfortunately, pharmacological agents have shown low specificity toward SLC1A5 [49]. In the context of HCC, competitive inhibition of glutamine entry poses a threat to adjacent non-tumoral liver, which is highly metabolically functional. Proline-derived inhibitors, such as gamma 4-biphenylmethyl-L-proline, have the potential of targeting SLC1A5 in a non-competitive manner and have been predicted to do so in silico [50,51]. Unfortunately, in mouse Dt81Hepa1-6 and human HCC cells, gamma 4-biphenylmethyl-L-proline was unsuccessful at inhibiting glutamine uptake (Figure S3). We consequently depleted SLC1A5 expression using siRNAs in Hep3B and Huh7 HCC cells. As expected, knockdown of SLC1A5 expression significantly decreased ^3^H-glutamine uptake in Huh7 and Hep3B cells, regardless of low or high availability of exogenous glutamine, suggesting that in HCC cells, SLC1A5 contributes to glutamine influx regardless of microenvironmental heterogeneity. We also evaluated the relative role of SLC38A2 in HCC glutamine consumption given that it was the most highly expressed transporter across the entire human HCC cell panel. Interestingly, SLC38A2 would ensure higher-affinity activity for glutamine, given that its loss of expression mainly impacted ^3^H-glutamine uptake when exogenous glutamine was scarce, which was consistently observed for both studied HCC models. Combined inhibition of SLC1A5 and SLC38A2 had the greatest effect on ^3^H-glutamine uptake, suggesting that these two transporters represent the bulk of glutamine consumption in HCC cells in a wide variety of cellular microenvironments. Under SLC38A2 siRNA treatment, in high glutamine conditions, but not glucose-replete conditions, Huh7 cells showed decreased ^3^H-glutamine uptake, whereas the opposite was observed for Hep3B cells. Such minor differences observed between both cell lines may arise from inherent subtleties in glutamine transporters that distinguish Huh7 and Hep3B cells. As both cells were independently isolated from individual tumors, such discrepancies may arise from HCC interindividual heterogeneity. Though loss of either SLC1A5 or SLC38A2, or both transporters, in HCC cells decreased ^3^H-glutamine uptake, residual uptake activity was detected, highlighting that other amino acid transporters are relevant in the flux of glutamine between HCC cells and their environment. Indeed, neither SLC1A5 nor SLC38A2 depletion affected HCC cell viability, nor did it negatively impact their ability to use exogenous glutamine for optimized survival. In addition, we compared the impact of genetically depleting SLC1A5 and SLC38A2 to pharmacological inhibition with GPNA, a small molecule inhibitor with broad selectivity for the amino acid transporters SLC38A2 and SLC1A5, but also SLC7A5, SLC7A8, SLC38A1, SLC38A4, and SLC38A5 [52]. Similar findings were observed under GPNA treatment: HCC cells maintained sufficient glutamine uptake to support dose-dependent utilization of extracellular glutamine for cell survival. When combined with SLC38A2, HCC cells became insensitive to GPNA treatment, suggesting that in HCC, SLC38A2 is a major target of GPNA despite being able to act on other glutamine transporters. Taken together, targeting glutamine transport in HCC will be a challenge because of the functional redundancy of amino acid transporters, even though knockdown of SLC1A5 and SLC38A2 did not lead to compensatory upregulation of other transporters at the mRNA level (Figure S2). Interestingly, in osteosarcoma 143B cells depleted of SLC1A5, Broer et al. observed compensation by SLC38A1 and SLC38A2 for glutamine transport, but not in triple-negative breast cancer HCC1806 cells [53]. Our data show that SLC1A5 and SLC38A2 would not be convincing targets for inhibiting glutamine uptake and subsequent glutamine-dependent metabolism in HCC. Also, targeting glutamine transporters may pose a threat to the metabolism of adjacent liver parenchyma, which is often already dysfunctional in cirrhotic tissue often associated with HCC.

In conclusion, with the positive effect of extracellular glutamine on murine and human HCC cell survival and proliferation, our findings suggest that there is an increased demand for glutamine by liver cancer cells. Indeed, glutamine uptake is greatly increased in murine HCC compared to normal hepatocytes. Hence, glutamine could be a limiting nutrient in liver cancer development and progression. Our comparative and comprehensive analysis of glutamine transporter has revealed a novel hallmark of HCC, characterized by a major reprogramming of transporter gene expression. Although blocking amino acid transporters in HCC is appealing for the targeting of glutamine addiction in liver cancer cells, extensive efforts will be required to determine if this is a viable approach in cancer treatment. Finally, as glutamine transporter expression profiles can stratify HCC patients according to their risk of mortality, they have robust potential of being prognostic biomarkers for the overall survival of patients suffering from HCC.

## 4. Materials and Methods

### 4.1. Reagents

Dulbecco’s modified Eagle’s medium (DMEM), i.e., high-glucose culture medium and glutamine/glucose/pyruvate-free medium, fetal bovine serum (FBS), penicillin/streptomycin, Leibovitz’s L-15 medium, TRIzol^®^ reagent, and L-Glutamine were purchased from Invitrogen (Burlington, ON, Canada). QuantiTect reverse transcription kit and QuantiTect SYBR Green PCR Kit were purchased from QIAGEN (Toronto, ON, Canada). (R)-gamma-(4-Biphenylmethyl)-L-proline was purchased from Carbosynth (Gardner, MA, USA), and MTT (3-(4,5-Dimethylthiazol-2-yl)-2,5-diphenyltetrazolium bromide) tetrazolium salt was purchased from Bioshop (Burlington, ON, Canada). Type IV collagenase was from Worthington Biochemical (Lakewood, NJ, USA). Gamma-p-nitroanilide (GPNA) was purchased from Adooq Bioscience LLC (Irvine, CA, USA). All other products were purchased from Sigma-Aldrich (Oakville, ON, Canada) unless stated otherwise.

### 4.2. HCC Cell Lines

Authenticated murine Hepa1-6, human HepG2, Hep3B, Huh7, SNU-387, SNU-398, SNU-475, SNU449, PLC/PRF/5, and SK-HEP-1 cells were purchased from the American Type Culture Collection (Manassas, VA, USA). Human JHH-2, JHH-4, JHH-5, JHH-6, and JHH-7 cells were obtained from the Japanese Collection of Research Biosources Cell Bank. Human JHH-1 cells were a gift from Dr. Burton Yang of the University of Toronto, and HLE cells were from Dr. Marco Salvatore of the Italian National Centre for Rare Diseases. The murine Dt81Hepa1-6 cell line was obtained through in vivo passaging of Hepa1-6 cells in C57BL/6 mice [54].

### 4.3. Hepatocyte Isolation

Primary hepatocytes were isolated from male C57BL/6 mice (20–22 g), purchased from Charles River (Saint-Constant, QC, Canada), using the two-step perfusion method [55]. Briefly, under general anesthesia, the abdominal cavity was opened, and the liver was perfused in situ via cannulation of the portal vein for 4 min at 37 °C with calcium-magnesium-free HEPES buffer, then for 7 min at 37 °C with a solution of type IV collagenase [30 mg/100 mL] and CaCl_2_ [10 mM] in calcium–magnesium-free HEPES buffer. Cells were filtered through a 74 µm strainer, followed by three two-minute centrifugations (44× *g*) in Leibovitz’s L-15 medium supplemented with 0.2% bovine albumin. Trypan blue staining was used to validate cell viability greater than 80%. Primary hepatocytes were seeded onto Petri dishes in culture medium supplemented with 10% FBS, which was replaced with fresh medium 4 h after plating.

### 4.4. Cell Culture Conditions

Cells were cultivated in culture medium (high-glucose DMEM) supplemented with penicillin/streptomycin and 10% FBS at 37 °C and 5% CO_2_. For experiments, cells were left to attach to standard polystyrene culture plates overnight before onset. A cellular confluence of 70% was aimed for, with 0.065 M/cm^2^ (primary hepatocytes), 0.195 M/cm^2^ (Dt81Hepa1-6), 0.114 M/cm^2^ (Hepa1-6), 0.050 M/cm^2^ (Hep3B and Huh7), and 0.146 M/cm^2^ (HepG2). For proliferation assays, plates were seeded with 30% less cells, and 15% less for transfection protocols. Cells were incubated with glutamine/glucose/pyruvate-free DMEM supplemented with variable glutamine [0–4 mM] or high-glucose culture medium. Cell viability was assessed after cell treatment with experimental culture media by incubating cells with MTT [0.5/1 mg/mL] for 3 h, after which media were removed and the formazan crystals were solubilized using dimethyl sulfoxide. Cell viability was calculated as being proportional to the optical density (540 nm) of samples measured in a BioTek Instruments spectrophotometer (Winooski, VT, USA). Cell doubling time was assessed over 48 h using the crystal violet staining method. Briefly, after cell attachment, cells were treated with experimental culture media for 24, 36, and 48 h prior to being fixed with formaldehyde [4.8%]. Monolayers were also fixed following overnight attachment, but prior to the addition of the experimental media as an initial time-point measurement. Cells were stained with crystal violet solution [0.1% (*w*/*v*), 200 mM MOPS, pH 6.0], lysed using 10% acetic acid, and counted as being proportional to optical density (570 nm) measured in a BioTek Instruments spectrophotometer (Winooski, VT, USA). Cell doubling time was calculated using an online tool (https://doubling-time.com/compute_more.php (accessed on 4 August 2023).

### 4.5. ^3^H-Glutamine Uptake Assay

Dt81Hepa1-6 and primary hepatocytes were seeded in 24-well plates prior to uptake assay in culture medium supplemented with 10% FBS. The day following cell attachment, the media were replaced with glutamine/glucose/pyruvate-free medium containing 20 nM L-[3,4-^3^H(N)]-Glutamine [1 µCi/mL] purchased from Perkin Elmer (Woodbridge, ON, Canada). Cells were allowed to uptake glutamine from 1 to 24 h after the media change. Glutamine uptake was stopped by the removal of the ^3^H-glutamine-containing media, followed by an hour-long incubation with 10% trichloroacetic acid at 4 °C. Cells were washed two additional times with 10% trichloroacetic acid. Monolayers were then dried using 100% methanol and lysed using lysis buffer [0.5M NaOH, 1 mM EDTA, 0.1% Triton X-100]. Lysates were then added to the Ultima Gold liquid scintillation cocktail (Perkin Elmer, Woodbridge, ON, Canada). Liquid scintillation counting was performed using a Tri-Carb 2800TR analyzer (Perkin Elmer, Woodbridge, ON, Canada). Counts were normalized over total lysate proteins measured by a Bradford assay.

### 4.6. Transient Knockdown of Gene Expression

Prior to transfection, low-passage (<20) cells were seeded in antibiotic-free culture medium supplemented with 10% FBS and left to adhere overnight. The following day, transfection media were prepared using Dharmafect 1 transfection reagent according to the manufacturer’s recommendations (Horizon Discovery, Waterbeach, UK). Briefly, targeting and non-targeting siRNA [25 nM] or siGLO green [50 nM] (Horizon Discovery, Waterbeach, UK) solutions and Dharmafect 1 solution were prepared in antibiotic-free culture medium and incubated for 5 min at room temperature. siRNA and Dharmafect 1 solutions were combined to allow the formation of transfection agent and siRNA complexes at room temperature for an additional 20 min, then supplemented with 5% FBS before replacing culture media with the transfection media. The following day, cellular viability was assessed by MTT assay, ^3^H-glutamine uptake was measured, and RNA was extracted, whereas media were replaced with fresh culture medium (5% FBS) for protein extraction performed 48 h after transfection. To assess the cell doubling time, cells were transfected and cultured for 24, 36, and 48 h. siRNA sequences are detailed in Table S1. siGLO green was used as a transfection indicator.

### 4.7. Animals and Experimental Hepatocarcinogenesis

Male C57BL/6 mice (20–22 g) were purchased from Charles River (Saint-Constant, QC, Canada) and fed ad libitum with regular chow feed. Induction of murine HCC using Dt81Hepa1-6 cells was performed as previously described [54]. Briefly, Dt81Hepa1-6 cells were trypsinized and suspended in saline with 0.25% albumin, prepared at 1M cells (200 µL) in syringes with 25G needles. Under general anesthesia, an incision was made in the left upper region of the abdomen and the spleen was delicately pulled out onto a saline-soaked gauze (37 °C). Slowly, the cellular suspension was injected into the splenic parenchyma and the needle was removed once the spleen regained its color. A droplet of Vetbound veterinary glue (3M, London, ON, Canada) was applied to the injection site, the spleen was returned to the abdominal cavity, and the incision was closed. Mice were kept for 21 days before sacrifice to allow the formation of HCC lesions. At the time of sacrifice, the liver was separated into tumoral specimens and adjacent non-tumoral (peritumoral) specimens. Additionally, mice not having undergone any surgical intervention were used as controls for normal liver specimens. Samples were snap-frozen and kept at −80 °C until further analysis. All animal procedures conformed with the Canadian Council on Animal Care and were approved by the “Comité institutionnel de protection animale du CHUM”.

### 4.8. Human Samples

Normal liver tissue samples and hepatocyte isolates were obtained from patients enlisted for non-HCC-related liver surgery. The main exclusion criterion for participation was the presence of primary liver disease. Written informed consent was obtained prior to surgery for participation in the “Banque de données (cliniques et biologiques) et d’échantillons biologiques associés à des fins de recherche sur les cancers hépatobiliaires et pancréatiques (#09.237)”. This research protocol conformed to the Declaration of Helsinki and was approved by the “Comité d’éthique de la recherche du Centre de recherche du CHUM”.

### 4.9. Gene Expression Analysis

RNA was isolated using the phenol–chloroform extraction method with TRIzol^®^ reagent following the manufacturer’s protocol. Liver samples were homogenized directly in TRIzol^®^ reagent. RNA concentration and purity were confirmed using te NanoDrop^TM^ 1000 (Thermo Fisher Scientific, Mississauga, ON, Canada), and mRNA extracts (250 ng) were reverse-transcribed into complementary DNA using the QuantiTect Reverse Transcription Kit. mRNA expression was then quantified through real-time PCR using the QuantiTect SYBR Green PCR Kit in a Real-Time Rotor-Gene 3000 Thermocycler (Corbett Research, Sydney, Australia) over 40 amplification cycles. All primer sequences are listed in Table S2. Relative mRNA using semiquantitative PCR was calculated using the delta–delta CT method with 3 reference genes for murine samples (PPIA, HPRT1, and H2AFZ) and 2 reference genes for human samples (S9 and HMBS) [56].

### 4.10. Western Blotting

Cells were pelleted and lysed using RIPA lysis buffer containing protease and phosphatase inhibitors (extraction buffer). Liver samples were homogenized directly in extraction buffer. Sample protein concentrations were quantified using the Bradford colorimetric method. Proteins (50/100 µg) were boiled prior to loading onto a 10% SDS-polyacrylamide gel for electrophoretic migration. Proteins were transferred onto PVDF membranes by wet electroblotting overnight (4 °C). Membranes were blocked in PBST with 5% skim milk for one hour at ambient temperature and washed. Proteins were then probed with anti-ASCT2 (1:1000 (V501), Cell Signaling, Whitby, ON, Canada) overnight at 4 °C, in PBST containing 1% milk. Membranes were washed, then incubated with HRP-conjugated anti-rabbit IgG (1:2000, BD Biosciences, San Diego, CA, USA) in PBST containing 5% milk at room temperature for one hour. β-actin was probed using anti-actin kit (1:10,000, Calbiochem, Merck KGaA, Darmstadt, Germany) in 1% milk at room temperature for two h, washed, and treated with HRP-conjugated anti-mouse IgM (1:20,000, Calbiochem, Merck KGaA, Darmstadt, Germany) in 5% milk at room temperature for one hour. Membranes were washed prior to chemiluminescence revelation using a Novex^TM^ ECL Chemiluminescent Substrate Reagent Kit (Invitrogen, Burlington, ON, Canada) according to the manufacturer’s protocol.

### 4.11. Survival Analysis

Analysis of HCC patient overall survival was performed using the KM-plotter database [57,58]. Survival of HCC patients (*n* = 364) was compared between high and low intratumoral mRNA expression cohorts of probed genes using RNAseq data from The Cancer Genome Atlas-Liver Hepatocellular Carcinoma (TCGA-LIHC) program. High and low expression cohorts were calculated using the most appropriate cutoff value determined between lower and upper quartiles. No patient stratification was performed. The prognostic value of tumor expression levels of glutamine transporter genes was analyzed through Kaplan–Meier plotting with “death” as the outcome. Kaplan–Meier curves between the low and high expression cohorts were compared using the log-rank method. The median survival of each cohort and comparative hazard ratio were calculated for each dataset.

### 4.12. Statistical Analysis

Data are presented as mean ± standard error (SEM). All data represented are from a minimum of three independent experiments (each in duplicate or quadruplicate) or of three individual cell samples, participants (normal human liver and hepatocytes), or mice for mRNA and protein analyses. Statistical analysis was performed using GraphPad Prism 9 (San Diego, CA, USA). Appropriate two-tailed statistical methods were used for each dataset using one-way analysis of variance (ANOVA), two-way ANOVA, and Tukey’s post-test for multiple comparisons as well as paired and unpaired *t*-tests. Statistical differences were considered significant if *p* < 0.05 (*: *p* < 0.05, **: *p* < 0.01, ***: *p* < 0.001, ****: *p* < 0.0001). All authors had access to the study data and have reviewed and approved the final manuscript.

## Data Availability

All data will be made available to inquirers following approval from the manuscript authors.

## Supplementary Materials

The following supporting information can be downloaded at: https://www.mdpi.com/article/10.3390/ijms25147558/s1.
