# Supplementary material for: Reprogramming of Glutamine Amino Acid Transporters Expression and Prognostic Significance in Hepatocellular Carcinoma
Source: Int J Mol Sci. 2024 Jul 10;25(14):7558. doi: 10.3390/ijms25147558 (PMC11277143; doi:10.3390/ijms25147558)
Supplement: Supplementary file 1 [file ijms-25-07558-s001.zip › ijms-3069448-supplementary.pdf]

Table S1. siRNA catalog numbers obtained from Dharmacon.

**siRNA**	**Dharmacon catalog number**
ON-TARGETplus Human SLC1A5 (6510) siRNA SMARTpool	L-007429-00-0005
ON-TARGETplus Human SLC38A2 (54407) siRNA SMARTpool	L-007559-01-0005
ON-TARGETplus Non-targeting siRNA pool D-001810-10-05	D-001810-10-05
siGLO Green Transfection Indicator	D-001630-10-05

Table S2. Primer sequences used for qPCR. m: mouse, h: human.

Gene			Sequence		Amplicon length
**mPPIA**	*F*	5'-	CGCGTCTCCTTCGAGCTGTTTG	-3'	150
	R	5'-	TGTAAAGTCACCACCCTGGCACAT	-3'	
**mHPRT1**	*F*	5'-	GCTTGCTGGTGAAAAGGACCTCTCGAAG	-3'	117
	R	5'-	CCCTGAAGTACTCATTATAGTCAAGGGCAT	-3'	
**mH2AFZ**	*F*	5'-	ACAGCGCAGCCATCCTGGAGTA	-3'	202
	R	5'-	TTCCCGATCAGCGATTTGTGGA	-3'	
**mSLC1A5**	*F*	5'-	CCATTCTTCTCCTCTACACACTTC	-3'	143
	R	5'-	CCTCTCATCTACTTCCTCTTCAC	-3'	
**mSLC6A14**	*F*	5'-	GTTTCTCCTTGGCCTCCTCT	-3'	84
	R	5'-	ATTCCCCATCCAGCACAGAA	-3'	
**mSLC7A6**	*F*	5'-	ATTGTTGAGCCCACCAGTCA	-3'	202
	R	5'-	GGACCTTGGCATACGTGAAT	-3'	
**mSLC38A1**	*F*	5'-	CCAAGCATGCGATGTAAGGG	-3'	164
	R	5'-	CTAACCGGAACGGAGACAGG	-3'	
**mSLC38A2**	*F*	5'-	ACTCATACCCCACCAAGCAG	-3'	204
	R	5'-	TGGCATAAGAAAGCCCAAGGA	-3'	
**mSLC38A3**	*F*	5'-	ATCTCACCCTCTGGAACGGA	-3'	71
	R	5'-	CTCTATATCCCCCGGCCGTA	-3'	
**mSLC38A5**	*F*	5'-	CAACCTCAGCAACGCTATCAT	-3'	112
	R	5'-	AGACAGGAGAGCAATGCACAG	-3'	
**hHMBS**	*F*	5'-	AAGCGGAGCCATGTCTGGTAAC	-3'	84/151/175
	R	5'-	GTACCCACGCGAATCACTCTCA	-3'	
**hS9**	*F*	5'-	CGTCTCGACCAAGAGCTGA	-3'	123
	R	5'-	GGTCCTTCTCATCAAGCGTC	-3'	
**hSLC1A5**	*F*	5'-	TCATGTGGTACGCCCCTGT	-3'	86
	R	5'-	GCGGGCAAAGAGTAAACCCA	-3'	
**hSLC6A14**	*F*	5'-	TATGGCGCAATTCCATACCC	-3'	175
	R	5'-	CCAGGTATGGACCCCAGTTA	-3'	
**hSLC7A6**	*F*	5'-	GCGCTCATTGCCATCATTGT	-3'	102
	R	5'-	GTTTCCCATGTCCCAGGAGG	-3'	
**hSLC38A1**	*F*	5'-	GCTTTGGTTAAAGAGCGGGC	-3'	151
	R	5'-	CTGAGGGTCACGAATCGGAG	-3'	
**hSLC38A2**	*F*	5'-	GCATCTGCAGCTTCTATGTTGA	-3'	119
	R	5'-	CCACTTAACAGGAAGAACAAAGC	-3'	
**hSLC38A3**	*F*	5'-	CATCACGCTCCAGAACATCG	-3'	112
	R	5'-	CCAGTCCGAGGTTTTCTCCT	-3'	
**hSLC38A5**	*F*	5'-	GAGAGGGTGCCCGAACCT	-3'	195
	R	5'-	CCTCGAAATCCATGAACTGGAC	-3'	
**hSLC7A5**	*F*	5'-	CATCCTGCTGGGCTTCGT	-3'	81
	R	5'-	AGTTTGGTGCCTTCAAATGAGAA	-3'	
**hGLUL**	*F*	5'-	TGCTGGTGTAGCCAATCGTA	-3'	354
	R	5'-	CCCTCTATCCCAGCCAAACA	-3'	
**hGLS1**	*F*	5'-	TCTACAGGATTGCGAACGTCT	-3'	100
	R	5'-	CTTTGTCTAGCATGACACCATCT	-3'	

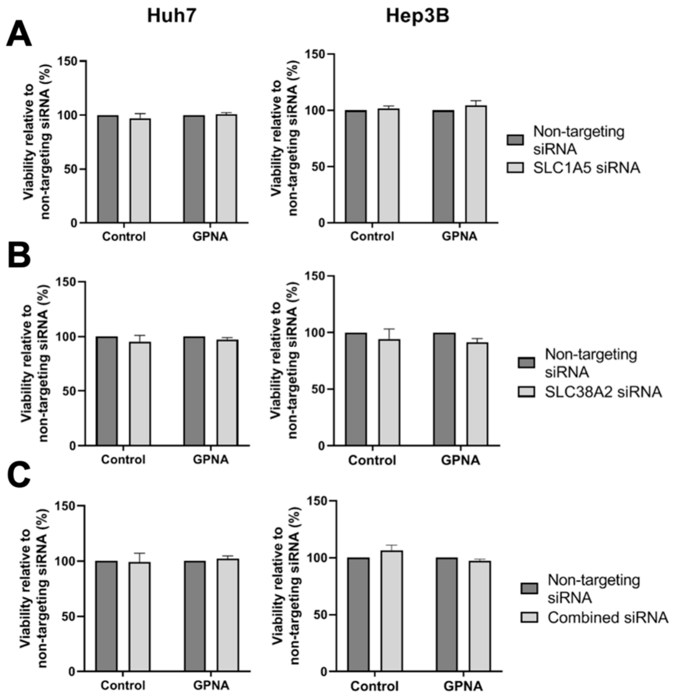

Figure S1. Molecular inhibition of SLC1A5 and SLC38A2 insufficiently harnesses HCC cell viability. siRNA transfection was targeted toward SLC1A5 (A), SLC38A2 (B), or both (C) glutamine transporters. After transfection, cells were conditioned to glucose/glutamine-rich media without (control) or with gamma-p-nitroanilide (GPNA) [0.5 mM]. Cell viability was assessed by MTT assay and represented as a percentage compared to non-targeting siRNA-treated Huh7 and Hep3B HCC cells.

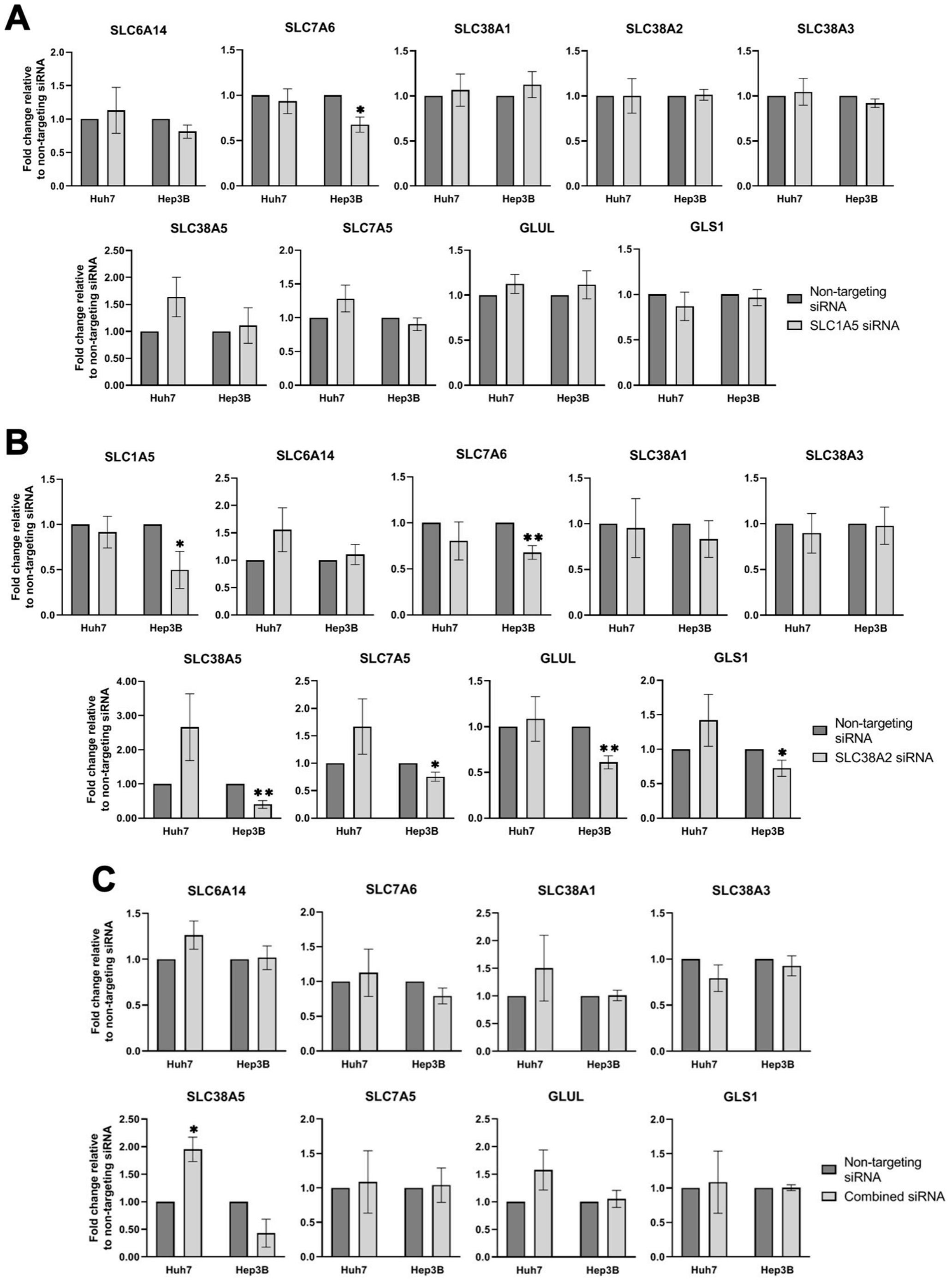

Figure S2. mRNA expression of alternative amino acid transporters and glutamine-related enzymes under SLC1A5 and SLC38A2 molecular knockdown. siRNA transfection targeted SLC1A5 (A), SLC38A2 (B), or both (C) glutamine transporters in Huh7 and Hep3B HCC cells. Relative mRNA expression for genes are represented as fold-change compared to non-targeting siRNA. SLC7A5: glutamine-essential amino acid exchanger LAT1, GLUL: glutamine synthetase, GLS1: glutaminase 1. *: $p < 0.05$, **: $p < 0.01$.

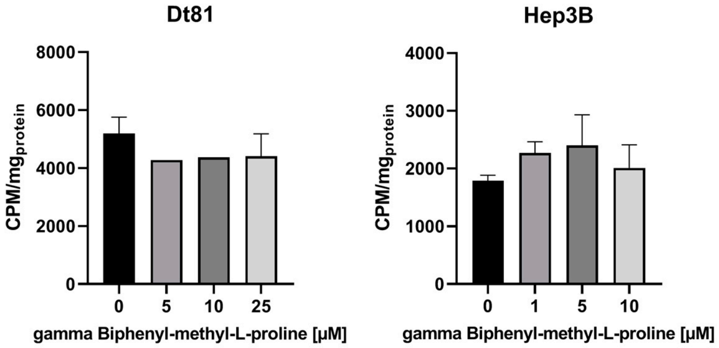

Figure S3. Gamma 4-biphenylmethyl-L-proline unsuccessfully inhibits glutamine uptake in HCC cells. Pharmacological inhibition of SLC1A5 using the gamma 4-biphenylmethyl-L-proline small molecule in Dt81Hepa1-6 (Dt81) and Hep3B HCC cells.
